# Commercial importance of seaweeds: an overview

**DOI:** 10.1186/s40643-025-00944-y

**Published:** 2025-10-27

**Authors:** Subhashree Rath, Subhashree Subhasmita Sahoo, Aurodeepa Samantaray, Pratikshya Parhi, Chita Ranjan Sahoo, Hrudayanath Thatoi

**Affiliations:** 1 Centre for Industrial Biotechnology Research, Sikha ‘O’ Anusandhan Deemed to Be University, Bhubanseswar, Odisha 751003 India; 2 Department of Health Research, Ministry of Health and Family Welfare, ICMR-Regional Medical Research Centre, Govt. of India, Bhubaneswar, Odisha 751023 India

**Keywords:** Marine ecosystem, Biochemical compound, Hydrocolloids, Sustainable, Nutraceuticals

## Abstract

Seaweeds, a vital source of marine ecosystems, are gaining popularity for their importance in ecological, economic, and industrial applications. These are profusely found in coastal areas due to the promising environmental factors. Various biochemical compounds, such as polysaccharides, proteins, lipids, and bioactive chemicals, are present in seaweed, which is useful in numerous industrial applications. Moreover, seaweeds are becoming a useful resource for addressing various environmental sustainability issues and food security, hydrocolloids to functional foods and nutraceuticals. Therefore, the cultivation and harvesting of seaweed using advanced and unique techniques are expanding globally for its efficient and sustainable production. In-depth understanding of the structural variation of seaweed, its taxonomy, and adaptation mechanisms is essential to evaluate its importance in the marine ecosystem. This review focused on botany, advanced farming techniques, and industrial prospects of seaweed, that especially emphasize the role of seaweed products in promoting a sustainable food industry. Further, this review also elaborates on various functional foods, bioactive compounds, and hydrocolloids derived from seaweed to improve food security and promote good health conditions. Besides this, insights into seaweed products for sustainable industrial application of seaweed are also emphasized in this review to address various issues related to global food systems, economic development, and environmental preservation.

**Graphical Abstract:**

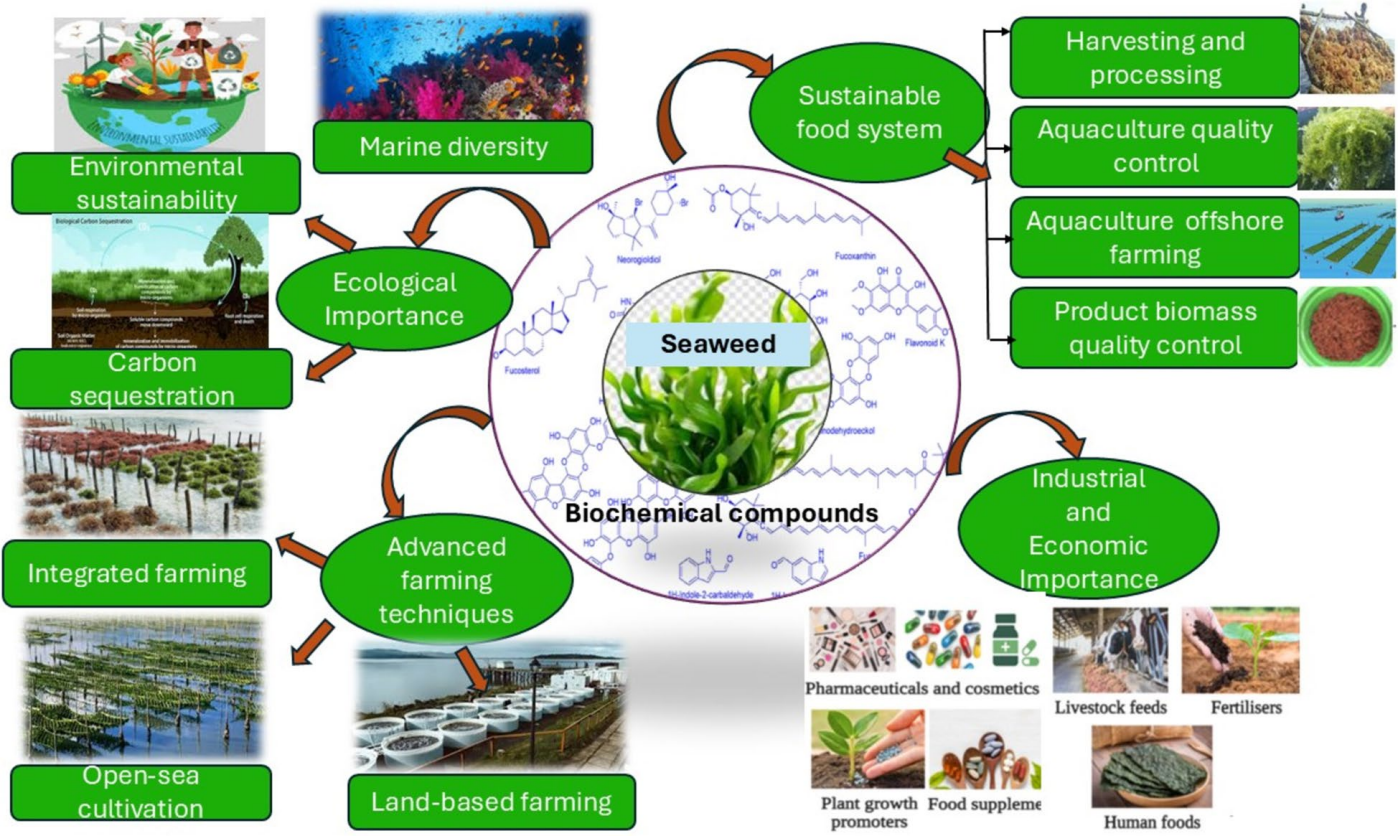

## Introduction

Seaweeds, also known as marine macroalgae, are among the most important and valuable marine source commodities. Seaweeds are extremely diverse creatures, with widely variable sizes, forms, and life cycles (Sobuj et al. 2024). According to their physical characteristics and pigmentation, these are mostly classified into three families, namely *Rhodophyta* (red algae), *Phaeophyta* (brown algae), and *Chlorophyta* (green algae) (Sobuj et al. 2024). Some seaweeds have adapted to protected coastal areas, while others thrive in exposed areas with heavy waves and tides (Mohammadigheisar et al. 2020). Different seaweed species are commonly used in their raw form as fertilisers to enhance agricultural soil fertility. Seaweeds are also used in manufacturing of medicine, human diets, and animal feed (Jamal et al. 2017). These seaweeds contain important trace elements like Fe, Mn, Cu, Zn, Co, Se, Br and I and a good source of K, Na, Ca, Mg, and P_4_ (Corino et al. 2019; Kumawat and Kumawat 2023). Additionally, some seaweeds are recognised for their nutrient bioavailability (Øverland et al. 2019). The bioactive compounds present in seaweeds are pigments, fatty acids, sterols, proteins, lipids, fibres, and polysaccharides, which have been shown to have good impacts on human health (Shahnaz et al. 2009; Cherry et al. 2019; Ioannou et al. 2009; Wells et al. 2017). More than 60 trace metals are significantly higher in concentration incase of seaweeds than terrestrial plants. The eminent mineral contents of seaweeds resulted from their capacity to take up inorganic materials from their surroundingsFurthermore, seaweeds also contain vitamins, essential amino acids, and antibiotics (Kumawat and Kumawat 2023).. Seaweeds contain 1–5% of lipids, however, the common of those lipids are PUFAs. The red and brown seaweeds have greater concentrations of PUFAs 20:5 n-3 (EPA) and 20:4 n-5 as compared to green algae (arachidonic acid) (Norziah and Ching 2000; Mišurcová et al. 2011; Mišurcová 2011). In coastal regions across the world, seaweed has long been consumed and is a staple food in many nations (Rahikainen et al. 2021). Seaweed biomass is a great substitute ingredient for cattle. Nowadays, consumers are more interested in nutrition and a healthy lifestyle and are looking for healthier food options (Alisha et al. 2019). There are about 600 different types of seaweed used for food globally. however, brown seaweed is the most popular, followed by red and green seaweed (Leandro et al. 2020). The Food and Agriculture Organisation of the United Nations have reported that over 200 species of macroalgae are valuable in Europe for their nutritional and commercial properties (L¨ahteenm¨aki-Uutela et al. 2021). Particularly in China, Japan, Korea and Southeast Asia, seaweeds have long been a mainstay of Asian cusines. Seaweeds are common organisms that mostly employ photosynthesis to transform CO_2_ into sugars and oxygen during daytime (Van Ginneken 2017). These are commonly eaten as fresh salads or cooked vegetables along with rice. Seaweeds are also used in the preparation of meat and fish curry, soup, porphyra, undaria, and laminaria dishes (Manickavasagam et al. 2019). India consists of more than 7500 km of coastline, which supports diverse marine flora and rich variety of seaweeds. The highest number of seaweed species was found in Indian islands like the Andaman and Nicobar and Lakshadweep and some coastal places of the southeast part of India, especially in Tamil Nadu and the Gulf of Mannar (Jaikumar et al. 2024). Specifically, the seaweeds like *Chlorophyceae*, *Phaeophyceae*, and *Rhodophyceae* algae were found in the tropical and subtropical coastal waters of India, which supply a suitable habitat for the development and growth of these seaweeds (Mantri et al. 2019). Apart from these seaweed species, some other industrially significant seaweeds such as *Ulva lactuca*, *Kappaphycus alvarezii*, *Sargassum*, *Turbinaria*, *Gracilaria*, and *Gelidiella* were also found in India (Ganesan et al. 2019). The seaweed industries of India are becoming more popular due to their significant importance in food, agriculture, biofuel production, nutraceuticals, and pharmaceuticals (Jaikumar et al. 2024). The increasing demand for seaweed-based products worldwide makes it more crucial than ever to comprehend the variety, distribution, and economic worth of seaweeds found along the Indian coast (Ganesan et al. 2019). Despite the increasing global and national interest, comprehensive information consolidating the taxonomy, distribution, biochemical potential, cultivation methods, and industrial applications of seaweeds found in India remains fragmented.

This review provides a complete insight of the commercially important seaweeds along with their distribution in India, taxonomy, chemistry, harvesting and cultivation techniques, impacts on the environment and socioeconomic, biochemical composition, nutritional value and the numerous viable industrial applications of these seaweeds. the potential for sustainable exploitation of seaweed resources, highlighting both the obstacles and opportunities in the seaweed industry.

## Botany

Seaweeds have a wide variety of morphology from basic filamentous structures to sophisticated thalli and are classified as both members of the kingdom Protista or Plantae. These are photosynthetic marine organisms that are found in different colours, sizes, and shapes, and play an important role in marine ecosystems (Andersen 1998). The seaweeds lack true roots, stems, or leaves. The main body part of seaweed is called the thallus, which is undifferentiated into roots, stems, or leaves (Phillips 2023). The thallus of seaweed is simple and filamentous or complex and large. The thallus can vary according to its size, like small and filamentous or large and complex (Baweja et al. 2016). Seaweeds show a variety of lengths depending on their morphologies and ploidy levels (Lobban and Wynne 1981). Seaweeds are green, red, and brown algae found in different regions worldwide. Green algae are closely connected to land plants (*Embryophytes*) in the *Archaeplastida* supergroup. These green algae have their place in the *Viridiplantae* clade as they have similarities with the common ancestor of terrestrial plants. Red algae are closely related to green algae and are classified under the supergroup *Archaeplastida*. Another seaweed brown algae belongs to kingdom Chromista and present in clade of *Stramenopiles*, which also includes diatoms and oomycetes. These are indistinctly linked with green and red algae and are not members of the supergroup *Archaeplastida* (Gololobova and Belyakova 2022). Some seaweed species are long-lived, like *Ascophyllum nodosum* and red algae (Frantz et al. 2005). The understanding of in-depth botany and exploration of the botany of new seaweed species is essential to fully understand the biodiversity of marine ecosystems and to maximise its potential in biotechnology and agricultural sectors.

## Distribution of seaweed

Seaweeds are found primarily in the coastlines of marine environments along with intertidal and subtidal zones. The distribution of seaweed is influenced by diverse environmental factors like temperature of water, availability of light in water, salinity, nutrient levels, and the nature of the substrate (e.g., rocky shores, sandy bottoms) (Kaliaperumal et al. 1995). Therefore, the diversity of seaweeds in intertidal zones decreases with the increase in height. There are around 11,000 species of seaweed known to exist in the world, including 1800 species of *Chlorophyceae*, 2000 species of *Phaeophyceae*, and 7200 species of *Rhodophyceae* (http://www.seaweed.ie). Some seaweeds were also found in distances in depths between 5.5 and 17 m, which showed a vertical gradient of beaches favourable for the growth of some species (Kaliaperumal et al. 1995). Seaweeds have been extensively grown for many years in 61 nations and territories, accounting for more than half of the production of marine and coastal aquaculture worldwide. Apart from these regions, seaweeds are reported to have its distribution in the Canary Islands and Lanzarote of the Atlantic Islands, islands of Europe, Columbo of South America, Africa, some regions of the Middle East, South-west Asia, South-east Asia, Asia and the South China Sea (Guiry and Guiry 2023). Seaweeds are basically can be found on the rocky shore regions of the Indian coastline like eastern coast of Visakhapatnam, Mahabalipuram, the Gulf of Kutch, Tuticorin, the Gulf of Mannar, Tiruchendur, southern coast of Kerala, Veraval, the Islands of Andaman and Nicobar, and Lakshadweep (Satheesh and Wesley 2012; Manikandan and Vivek 2024; Sahoo 2010). Apart from these places, seaweeds are also abundantly found in the areas surrounding Mumbai, Ratnagiri, Goa, Tamil Nadu (Karwar, Varkala, Vizhinjam, and Pulicat), and Odisha (Chilika) (Chennuri et al. 2019). Around 841 taxa of marine algae were discovered both in intertidal and deep-water locations throughout the coast of India. In India, 216 types of green seaweed, 434 types of red seaweed, and 194 types of brown seaweed have been discovered till the year 2019 (Mantri et al. 2019). Some of these species are economically important. Seaweeds play an important role in sea ecosystems like kelp forests and coral reefs, and act as chief producers and engineers of the ecosystem (Islam et al. 2021). These are economically significant because they serve as the basis for an effective food web (Hurtado et al. 2019). Six species of *Chlorophyceae* and two species of *Rhodophyceae* were discovered in the Sundarban Biosphere Reserve of West Bengal, India (Yadav et al. 2020). Moreover, ten macroalgae species, such as three Chlorophyta species, five *Phaeophyta* species, and *Rhodophyta* with two Rhodophyta species (*Digenea simplex* and *Actinotrichia fragilis* were found in the sandy shore of the Red Sea, Egypt (Farghali et al. 2023).

## Seaweed and chemistry

**Fig. 1:**
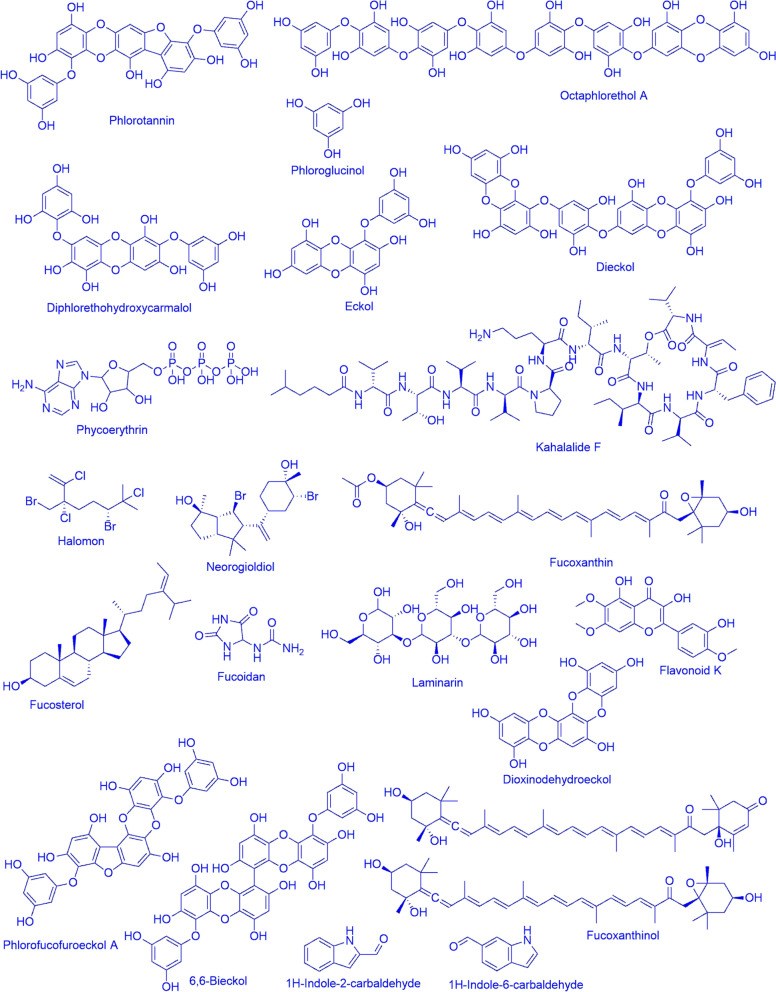
Important secondary metabolites found in Seaweeds

Seaweeds contain minerals, polysaccharides, vitamins, and bioactive substances like proteins, lipids, and polyphenols (Matanjun et al. 2009) and also have hypolipidemic properties (Chan and Matanjun 2017). Seaweeds are composed of 80–90% water on a wet weight basis. Depending upon the species and various environmental conditions, seaweeds contain 50% carbohydrates, 1–3% lipids, and 7–38% minerals on a dry weight basis. It has been found that the protein composition of seaweeds ranged from 10–47%, with a large proportion of important amino acids (García-Casal et al. 2007). Carbohydrate content and quantity differ across seaweed species. Red seaweeds consist of unique carbohydrates, including floridean starch (semi-amylopectin-like α-1,4 and α-1,6 linked glucan), cellulose, xylan, and mannan. In addition, sulfur-containing galactans like agar and carrageenan were also found in seaweed (Table 1, Fig. 1) (Jiménez-Escrig and Sánchez-Muniz 2000). Brown seaweed variants contain fucoidan, laminaran (β-1,3-glucan), cellulose, alginates, and mannitol (Fig. 1). The fibres of brown seaweed are particularly cellulose and insoluble alginates like Ca, Mg, or Na salts of alginic acid (1,4-linked polymer of β-D mannuronic acid and α-L-guluronic acid) (Silva et al. 2005). Moreover, seaweeds contain a total 33–50 g/100 g d.w. dietary fibre (Rupérez and Saura-Calixto 2001). Seaweeds are rich in calcium, making them an essential vegetable source. Calcium levels in chalky seaweed, lithothamnion, can reach 7% in dry weight and up to 25–34%. Consuming seaweed may benefit pregnant women, teenagers, and the elderly who are at a risk of calcium deficiency (Burtin 2003). Moreover, Significant fluoride concentrations of 19.17–53.70 mg/g have been found in Egyptian seaweeds (Masoud et al. 2004). Seaweeds have a substantial stock in coastal waters, making them a promising source of functional lipids despite having far lower lipid levels than marine fish. A recent study on brown seaweed found that levels of total lipid (TL) and omega-3 polyunsaturated fatty acids (PUFAs) change with the seasons.this study showed 15% TL per dry weight (DW) and more than 40% of omega-3 PUFAs per total fatty acids of the seaweed (Nomura et al. 2013). Seaweed species have numerous biological activities and are abundant source of bioactive chemicals. Brown seaweed lipids include various bioactive substances, such as omega-3 polyunsaturated fatty acids, omega-6 arachidonic acid, fucoxanthin, fucosterol, and some polyphenols. As it reveals various physiological benefits based on distinct molecular pathways, a prominent carotenoid fucoxanthin, in brown seaweeds, was thought to be a unique nutraceutical chemical (Miyashita et al. 2011). The bioactive compound fucoidans were produced by numbers of brown seaweeds such as *Sargassum fusiforme*, *Cladosiphon okamuranus*, *Adenocystis utricularis*,, *Undaria pinnatifida, Chnoospora minima*, *Sargassum hemiphyllum*, and *Spatoglossum asperum* which showed anticoagulant, anti-inflammatory, antiadhesive, antiangiogenic, anticancer, cardioprotective, anti-proliferative, gastric protection, anti-prion, anti-retroviral, immunostimulatory, anti-allergy, anti-tumor, anti-viral, and antimicrobial activity. Another brown seaweed *Fucus vesiculosus* produces fucoidans, Trifucodiphlorethol A, Trifucotriphlorethol A, and Fucotriphlorethol A which is useful in the preparation of pharmaceutical products against disease, Chemopreventive, Anti-atopic dermatitis, Anti-inflammatory, Anti-obesity (Table 1) (Cong et al. 2016; Haneji et al. 2005; Trinchero et al. 2009;; Kawashima et al. 2012; El-Beltagi et al. 2022; Hwang et al. 2011; Palanisamy et al. 2017). Apart from brown seaweeds, red seaweed/algae such as *Chondrus ocellatus* and *Stenogramme interrupta*, are renowned for their content of carrageenan, which contributes to their anti-tumor, and antiviral properties (Zhou et al. 2004; Cáceres et al. 2000). Moreover, the green algae (Ulvophyceae) like *Ulva rigida*, *Udotea flabellum*, *U. conglutinata*, *Caulerpa microphysa*, and *Halimeda tuna* are significant sources of vitamins, sulfolipids, terpenoids, and phycobilins with antihypertensive, antibacterial, ACE-inhibitory, and cytotoxic activities (Paiva et al. 2016; Fenical et al. 1984; Ho and Kuo 2024; Indira et al. 2013; Husni et al. 2024). Additionally, a yellow-green alga *Tribonema minus*, and lesser-known species like *Porphyra yezoensis*, and*Porphyra tenera*, have pharmacological activity with unique compounds such as porphyrans, phycoerythrins, octoplorethol A, and various vitamins (Table 1) (Chen et al. 2019; Qu et al. 2010; Charoensiddhi et al. 2017). These results demonstrate the broad applicability of seaweed-derived bioactives in the pharmacological, nutraceutical, and cosmeceutical domains, confirming their enormous therapeutic potential. L-Carrageenans, K-carrageenans, I and ε- carrageenans were produced by numbers of seaweeds such as *Schizymenia binderi, Chondrus ocellatus, Sargassum patens, Gigartina pistillata, Eucheuma* sp., and *Chondrus* sp. that showed antiviral activity against HSV-1, HSV-2, antitumoral activity on H-22 cells, antitumoral activity on colorectal cancer stem cells, and antiproliferative activity on HeLa cells, mammary cells, and fibroblasts respectively (Table 1) (Matsuhiro et al. 2005; Zhou et al. 2006; Cotas et al. 2020; Ali et al. 2021). The seaweeds such as *Ecklonia kurome, Ecklonia cava, Dictyota humifusa, Sargassum hemiphylum, Eisenia bicyclis, Cystoseira nodicaulis, Eisenia arborea, Ecklonia stolonifera, Bifurcaria bifurcate*, *Sargassum muticum, Sargassum hemiphyllum, Bifurcaria bifurcate, Ecklonia kurome, Cystoseira nodicaulis* produced phlorotannins (Table 1) (Nagayama et al. 2002; Yoon et al. 2009; Stirk et al. 2007; Na et al. 2005; Shibata et al. 2002; Ferreres et al. 2012; Sugiura et al. 2008; Lee et al. 2013; Montero et al. 2016; Gonçalves-Fernández et al. 2019; Na et al. 2005; Palanisamy et al. 2017; Ferreres et al. 2012). Apart from the bioactive compounds, seaweeds are chief source of hydrocolloids such as alginate and agar–agar. Alginate is used as an emulsifier, biocompatible thickening agent, non-toxic gel, and biodegradable stabilization agent in the food and pharmaceutical industries (Steen 2022). In the medical and pharmaceutical field, alginate is used in making drug carrier for control and targeted drug delivery (Lee and Mooney 2012). Another form of alginate i.e. alginate microsphere are used for the loading and releasing of chemo pharmaceutical. Alginate is used in making drug carriers for controlled trypsin and lysozyme. The seaweeds such as *Laminaria hyperborea*, *Laminaria digitata*, *Ascophyllum nodosum*, *Macrocystis pyrifera*, *Durvillaea potatorum*, *Ecklonia radiata*, *Sargassum* spp., and *Fucus* spp (Qin et al. 2008; Fertah et al. 2017; Steen 2022; Lee and Mooney 2012; Moen et al. 1999; Lorbeer et al. 2017; Westermeier et al. 2012; Kumar and Sahoo 2017; Kennedy et al. 2024;). Prominently used to extract alginate from it (Table 2). Its concentration varies across species, ranging from 10% in *Sargassum turbinaria* to as high as 55% in *Durvillaea potatorum* (Table 2). Alginate can absorb UV-rays, repair sun damage, useful in skin smoothing cream and renew small cells. Coloured products used in various textile industries were also produced using alginate. This colour compound was cleaner easier and cheaper than another chemical colour compound. The salts of sodium and potassium alginate was used in the formulation of animal feeds (Reddy et al. 2021).

**Table 1:** Commercially important seaweed species, their major bioactive compounds, and applications

Sl. No	Seaweed	Class	Family	Major bioactive compound/biopolymer	Application	References
1.	*Schizymenia binderi*	Florideophyceae	*Gigartinaceae*	carrageenans	Antiviral activity against HSV-1, HSV-2	Matsuhiro et al. 2005
2	*Ecklonia kurome*	Phaeophyceae	*Lessoniaceae*	Phlorotannins	Antimicrobial activity against *Campylobacter fetus*, *E. coli*, *Salmonella enteritidis*, *S. typhimurium*, *Vibrio parahaemolyticus*, and *Bacillus cereus*	Nagayama et al. 2002
3	*Chondrus ocellatus*	Florideophyceae	*Gigartinaceae*	L-carrageenans	Antitumoral activity on H-22 cells	Zhou et al. 2006
4	*Sargassum patens*	Phaeophyceae	*Sargassaceae*	Sulphate PSs	Antiviral activity against HSV-1, HVS-2	Zhu et al. 2003
5	*Ishige okamurae*	Phaeophyceae	*Ishigeaceae*	Fucoxanthin	Antitumoral activity on B16-F10 cells	Kim et al. 2013
6	*Caulerpa racemosa*	Ulvophyceae	*Caulerpaceae*	Phenols	Antiproliferative activity on Huh-7, HeLa cells	Tanna et al. 2020
7	*Laminaria Jaopnica*	Phaeophyceae	*Laminariaceae*	fucoxanthin	Antitumoral activity on lung cancer cells	Mei et al. 2017
8	*Champia parvula*	Florideophyceae	*Champiaceae*	Sulphated polysaccharides	Antitumoral activity on sarcoma 180 ascites cells	Lins et al. 2009
9	*Chondrus crispus*	Florideophyceae	*Gigartinaceae*	polysaccharide	Antimicrobial activity against *Salmonella Enteritidis*	Kulshreshtha et al. 2020
10	*Ulva Lactuca*	Ulvophyceae	*Ulvaceae*	Phenols	Antiproliferative activity on MCF-7, HeLa cells	Saeed et al. 2020
11	*Gigartina pistillata*	Florideophyceae	*Gigartinaceae*	i- and ε- carrageenans	Antitumoral activity on colorectal cancer stem cells	Cotas et al. 2020
12	*Ecklonia cava*	Phaeophyceae	*Lessoniaceae*	Phlorotannins (7-phloroeckol)	UV photoprotection in B16F10 melanoma cells	Yoon et al. 2009
13	*Dictyota humifusa*	Phaeophyceae	*Dictyotaceae*	Phlorotannins	AChE-inhibitory activity	Stirk et al. 2007
14	*Sargassum hemiphylum*	Phaeophyceae	*Sargassaceae*	Phlorotannins	Treatment of atopic dermatitis	Na et al. 2005
15	*Halimeda tuna*	Ulvophyceae	*Halimedaceae*	Diterpene	Antiviral activity against corona virus strain A5Y	Koehn et al. 1991
16	*Gracilaria dominguensis*	Florideophyceae	*Gracilariaceae*	Agar-type PSs	Anticancer activity on EAC cells	Fernández et al. 1989
17	*Eisenia bicyclis*	Phaeophyceae	*Lessoniaceae*	Phlorotannins (PFFA, dieckol, eckol, bieckol)	Hyaluronidase	Shibata et al. 2002
18	*Caulerpa racemosa*	Ulvophyceae	*Caulerpaceae*	Caulerpin	Antiviral activity against HSV-1	Macedo et al. 2012
19	*Ulva fasciata*	Ulvophyceae	*Ulvaceae*	Phenols	Antiproliferative activity on PC3, HepG2 cells	Saeed et al. 2020
20	*Eucheuma* sp., *Chondrus* sp.	Florideophyceae,	–	κ -carrageenans	Antiproliferative activity on HeLa cells, mammary cells, and fibroblasts	Ali et al. 2021
21	*Cystoseira nodicaulis*	Phaeophyceae	*Sargassaceae*	Phlorotannins	Hyaluronidase-inhibition activity	Ferreres et al. 2012
22	*Eisenia arborea*	Phaeophyceae	*Lessoniaceae*	Phlorotannins	Antiallergic effects	Sugiura et al. 2008
23	*Ecklonia stolonifera*	Phaeophyceae	*Lessoniaceae*	Phlorotannins(dieckol)	Antimicrobial activity against *Staphylococcus aureus*	Lee et al. 2013
24	*Bifurcaria bifurcate*, *Sargassum muticum*	Phaeophyceae	*Sargassaceae*	Phlorotannins	Antitumoral activity on HFF-1, MKN-28, HT-29, Caco-2, BEL-7402, P388, ATDC5 cells	Montero et al. 2016, Gonçalves-Fernández et al. 2019
25	*Caulerpa sp.*	Chlorophyceae	–	Caulerpin	Antimicrobial activity against *Mycobacterium tuberculosis*	Canche Chay et al. 2014
26	*Ulva fasciata*	Ulvophyceae	*Ulvaceae*	Phenols	Antimicrobial activity against *Klebsiella pneumoniae*, *Proteus mirabilis*, Antifungal activity against *Aspergillus niger*, *A. sfumingatus*, *A. flavus*	Saeed et al. 2020
27	*Cystoseira tamariscifolia*	Phaeophyceae	*Sargassaceae*	Phenols	AChE-inhibitory activity	Custódio et al. 2016
28	*Sargassum hemiphyllum*	Phaeophyceae	*Sargassaceae*	Phlorotannins	Treatment of atopic dermatitis	Na et al. 2005
29	*Spatoglossum asperum*	Phaeophyceae	*Dictyotaceae*	Sulphated polysaccharide	Showing antimicrobial activity against *Aeromonas hydrophila*	Palanisamy et al. 2017
30	*Cladosiphon okamuranus*	Phaeophyceae	*Chordariaceae*	Fucoidan	Showing antimicrobial activity against Helicobacter pylori	Shibata et al. 2003
31	*Fucus vesiculosus*	Phaeophyceae	*Fucaceae*	Fucoidan, Trifucodiphlorethol A, Trifucotriphlorethol A, Fucotriphlorethol A	Ameliorated ear swelling, improved abdomen *skin lesions*, and decreased *inflammatory cell* infiltration	Tian et al. 2019
32	*Sargassum fusiforme*	Phaeophyceae	*Saragassaceae*	Fucoidan	Inhibit the formation of the tube and the migration of human microvascular endothelial cells and act as potent anti-angiogenic agent	Cong et al. 2016
33	*Cladosiphon okamuranus*	Phaeophyceae	*Chordariaceae*	Fucoidan	The proliferation of HTLV-1-infected T-cell lines and ATL patients' peripheral blood mononuclear cells was markedly suppressed by fucoidan, but not that of healthy peripheral blood mononuclear cells	Haneji et al. 2005
34	*Adenocystis utricularis*	Phaeophyceae	*Adenocystaceae*	Fucoidan	It showed potent anti-HIV-1 activity both against WT and drug-resistant HIV-1 strains	Trinchero et al. 2009
35	*Undaria piaantifida*	Phaeophyceae	*Alariaceae*	Fucoidan	Immunostimulatory, Anti-allergy, Anti-tumor, Anti-viral,	Kawashima et al. 2012
36	*Chondrus ocellatus*	Florideophyceae	*Gigartinaceae*	Carrageenan	Anti-tumor	Zhou et al. 2004
37	*Ishige okamurae*	Phaeophyceae	*Ishigeaceae*	Diphlorethohydroxycarmalol	Anti-diabetic,Anticancer,Radioprotective, Antioxidant, Photoprotective, Antityrosinase, Antimelanogenic,Neuroprotective	Lee et al. 2012
38	*Spartina patens*	Phaeophyceae	*Poaceae*	2-(4-(3,5 dihydroxyphenoxy)-3,5-dihydroxyphenoxy) benzene-1,3,5-triol (DDBT),	Anti-diabetic	Kawamura-Konishi et al. 2012
39	*Stenogramme interrupta*	Rhodophyceae	*Florideophyceae*	Carrageenan	Anti-viral	Cáceres et al. 2000
40	*Porphyra yezoensis*	Bangiophyceae	*Bangiaceae*	Porphyrans, Vitamin B2, B12, Choline	Antihypertensive, Anticoagulant,	Qu et al. 2010
41	*Ulva rigida*	Ulvophyceae	*Ulvaceae*	Ascorbic acid, Vitamin E, Carotene, Sulfolipid	Antihypertensive	Paiva et al. 2016
42	*Udotea flabellum*	Ulvophyceae	*Udoteaceae*	Udoteafuran, Udoteatrial	Antibacterial,	Fenical et al. 1984
43	*U. conglutinata*	UTC clade	*Udoteaceae*	Flexilin	Antimicrobial	Fenical et al. 1984
44	*Laurencia dendroidea*	Florideophyceae	*Rhodomelaceae*	Elatol	Acaricidal and repellent activity, Antileishmanial activity	Born et al. 2012
45	*Griffithsia* sp.	Florideophyceae	*Wrangeliaceae*	Griffithsin (protein)	Antiviral activity against MERS-CoV virus and SARS-CoV glycoprotei	Millet et al. 2016
46	*Gracilaria edulis*	Florideophyceae	*Gracilariaceae*	Flavonoid	Hypoglycemic activity	El-Beltagi et al. 2022
47	*Padina tetrastromatic*	Phaeophyceae	*Dictyotaceae*	Carrageenan	Anti-inflammation	Mohsin and Kurup 2011
48	*Tribonema minus*	Xanthophyceae	*Tribonemataceae*	Carrageenan	Anticancer activity	Chen et al. 2019
49	*Sargassum hemiphyllum*	Phaeophyceae	*Saragassaceae*	Fucoidan	Anti-inflammation	Hwang et al. 2011
50	*Fucus evanescens*	Phaeophyceae	Fucaceae	Fucoxanthins	Antiviral activity against ECHO-1, HIV-1, HSV-1, HSV-2	Krylova et al. 2021
51	*Caulerpa microphysa*	Ulvophyceae	*Caulerpaceae*	Terpenoids	Angiotensin I-converting enzyme (ACE) inhibitor Anti-tumor	Ho and Kuo 2024
52	*Pterocladia capillacea*	Florideophyceae	*Pterocladiaceae*	Ethyl 5,8,11,14,17-icosapentaenoate, Phytol	Antioxidant, Antibacterial	Charoensiddhi et al. 2017
53	*Lobophora variegata*	Phaeophyceae	*Dictyotaceae*	Porphyra-334	Antiviral activity	Ryu et al. 2014
54	*Durvillaea antarctica*	Phaeophyceae	*Durvillaeaceae*	Omega-3, Omega-6 Fatty acid	Moisture retention	Kalasariya et al. 2021
55	*Gongolaria nodicaulis*	Phaeophyceae	*Sargassaceae*	1,3,5-trihydroxybenzene	Hyaluronidase inhibition antiaging	Kalasariya et al. 2021
56	*Schizymenia dubyi*	Florideophyceae	*Schizymeniaceae*	Sulfated glucuronogalactan	Tyrosinase inhibition, Anti-HIV	Kalasariya et al. 2021 and Khalid et al. 2018
57	*Sargassum silauastrum*	Phaeophyceae	*Sargassaceae*	Polyphenols, Terpenoids, Fucoxanthin, Fatty acids	Tyrosinase inhibition, Anti-inflammation	Kalasariya et al. 2021, Saraswati et al. 2019
58	*Spatoglossum asperum*	Phaeophyceae	*Dictyotaceae*	Fucoidans	Antimicrobial activity	Palanisamy et al. 2017
59	*Bifurcaria bifurcata*	Phaeophyceae	*Sargassaceae*	Phlorotannins	Antitumoral activity	Palanisamy et al. 2017
60	*Ecklonia kurome*	Phaeophyceae	*Lessoniaceae*	Phlorotannin	Antimicrobial activity	Nagayama et al. 2002
61	*Ishige okamurae*	Phaeophyceae	*Ishigeaceae*	Fucoxanthin	Antitumoral activity	Kim et al. 2013
62	*Padina australis*	Phaeophyceae	*Dictyotaceae*	Phenols	Antimicrobial activity	Chellappan et al. 2020
63	*Cystoseira nodicaulis*	Phaeophyceae	*Sargassaceae*	Phlorotannins	Hyaluronidase-inhibition activity	Ferreres et al. 2012
64	*Halimeda tuna*	UTC clade	*Halimedaceae*	Phycobilin, Diterpenetrialdehyde	Antimicrobial activity, Antioxidant activity, Cytotoxicity activity	Indira et al. 2013 and Husni et al. 2024

**Table 2:** Application of hydrocolloids derived from seaweed

Name of the hydrocolloids found in seaweed	Name of seaweed	Chemical classification	Bioactivity	Concentration of hydrocolloids (%)	Application	References
Alginate	*Laminaria hyperborea*	Anionic polysaccharide	Biocompatibility, Non-toxicity, biodegradability, Gel forming nature	16	Biomedical, pharmaceutical, Wound healing, cell transplantation delivery of bioactive agents	Lee and Mooney 2012
Alginate	*Laminaria digitata*	Anionic polysaccharide	Biocompatibility, Non-toxicity, biodegradability, Gel forming nature	22–30	Drug delivery micro- and nanoparticles	Qin et al. 2008; Fertah et al. 2017
Agar agar	*Pterocladia capillacea*	polysaccharide	Gel forming, biodegradibility	43%		Rao and Bekheet 1976
Alginate	*Ascophyllum nodosum*	Anionic polysaccharide	Biocompatibility, Non-toxicity, biodegradability, Gel forming nature	25–44	Biostimulant, salt preservation	Smidsrod et al. 1990; Moen et al. 1999; Shukla et al. 2019
Alginate	*Macrocystis pyrifera*	Anionic polysaccharide	Biocompatibility, Non-toxicity, biodegradability, Gel forming nature	46.8	Bioplastic, food preservation, and products of the pharmaceutical industry	Lorbeer et al. 2017
Alginate	*Durvillaea potatorum*	Anionic polysaccharide	Biocompatibility, Non-toxicity, biodegradability, Gel forming nature	55	Agriculture, food, and pharmaceutical industries	Lorbeer et al. 2017
Alginate	*Ecklonia radiata*	Anionic polysaccharide	Biocompatibility, Non-toxicity, biodegradability, Gel forming nature	44	Production of biostimulants, extraction of phlorotannins and laminarin, human and animal product	Lorbeer et al. 2017; Nepper-Davidsen et al. 2023
Alginate	*Sargassum wightii*	Anionic polysaccharide	Biocompatibility, Non-toxicity, biodegradability, Gel forming nature	33	Emulsifier, thickener, stabilizer, viscosifier, gelling agent, therapeutic agent. film former in pharma and food industries	Kumar and Sahoo 2017; Kennedy et al. 2024
Alginate	*Sargassum multican*	Anionic polysaccharide	Biocompatibility, Non-toxicity, biodegradability, Gel forming nature	25.6	Packaging, bioplastic, production of film in the pharma industries	Belattmania et al. 2020; Mohammed et al. 2023
Alginate	*Laminaria ochroleuca*	Anionic polysaccharide	Biocompatibility, Non-toxicity, biodegradability, Gel forming nature	27.5	Biotechnology and medical applications	Belattmania et al. 2020; Kaidi et al. 2022
Alginate	*Cystoseina humilis*	Anionic polysaccharide	Biocompatibility, Non-toxicity, biodegradability, Gel forming nature	19.1	Packaging, bioplastic, production of film in the pharma industry	Belattmania et al. 2020
Alginate	*Fucus vesiculosus F. volubilis*	Anionic polysaccharide	Biocompatibility, Non-toxicity, biodegradability, Gel forming nature	18.3	Packing, bioplastic and in food industries	Belattmania et al. 2020
Alginate	*Carpodesmia tamariseifolia*	Anionic polysaccharide	Biocompatibility, non-toxicity, biodegradability, gel-forming nature	17.22	Biotechnology and food industry	Belattmania et al. 2020
Alginate	*Sargassum turbinaria*	Anionic polysaccharide	Biocompatibility, non-toxicity, biodegradability, gel-forming nature	10	Biotechnology	Fenoradosoa et al. 2010
Alginate	*Gracilaria folifera*	polysaccharide	Gel forming, biodegradability	20–23%	Biotechnology and food industry	Matsuhashi and Hayashi 1972
Agar agar	*Gracilaria edulis*	*Polysaccharides*	Food grade gel	6–8%	Food industry	Kaliaperumal and Uthirasivan 2001

## Cultivation and harvesting techniques

The need for food grain production is increasing due to population growth, which makes it more important than ever to manage natural resources efficiently (Jagtap et al. 2022). Seaweed farming, a new field of food production, has the potential to address the world's expanding food needs. Seaweed-derived hydrocolloids are valuable raw materials in industries such as health, food, medicines, textiles, fertilisers, and animal feed. Hydrocolloids and their derivatives play a significant role in the preservation and preparation of foods (Tanna and Mishra 2018; Yan et al. 2020). Asia is a key player in global seaweed production. Seven out of top ten seaweed-producing countries are located in Asia. Asian countries produce 99.1% of seaweeds for food industries (Behera et al. 2022). Seaweed cultivation is important as a source for meeting future energy needs. It also provides a clean solution by developing algae biorefineries coupled with seaweed farming. Furthermore, growing seaweed alongside algae aids coral reef conservation by encouraging growth and providing home for a variety of marine species (Hasselström et al. 2018). Seaweed mitigates eutrophication by sequestering excess nutrients from acquatic environments and generating oxygen as a byproduct (Behera et al. 2022; Krag et al. 2015). Moreover, cultivation of seaweed offers several direct and indirect advantages, along with creating opportunities for the employment coastal communities. As an environmentally beneficial method, it lessens the demand on natural land-based resources, guarantees a consistent source of seaweed biomass for several industrial applications, and aids reduction of pollution in coastal regions (Jagtap et al. 2022).

## Principle involved in seaweed farming

The potential of farmed seaweeds as a beneficial addition to crops has been shown by the notable rise in both output and demand. Large tracts of sea may be transformed into environmentally friendly agricultural fields in addition to expansive shoreline areas. Such large-scale production would provide enormous quantities of seaweed biomass in a controlled setting for a variety of industrial purposes using current technologies and cultural beliefs (Chopin 2015; Radulovich et al. 2015). Seaweed can be grown in the presence of sunlight, nutrients, O_2_, and CO_2_. Seaweed farming is an important process of cultivation in seawater to promote growth and encourage constant photosynthesis (Milledge et al. 2016). Several principles are found in the farming of seaweed, such as:

1. Selection of a suitable location
2. Selection and Preparation of species
3. Effective cultivation techniques
4. Maintenance of Field
5. Harvesting and drying

The most crucial aspect of seaweed farming is site selection (Yulianto et al. 2017). The Turtles and sea urchins affect farmers and physically damage their property. Furthermore, seaweed cultivation is hampered by illnesses like “ice-ice” (Egan et al. 2014). Hence, developing new strains of marine algae that are disease-resistant, lightweight, and heat-resistant is essential to tackle these challenges. Furthermore, more resilient and cost-effective agricultural methods need to be created, particularly for offshore environments (Radulovich et al. 2015).

## Types of seaweed cultivation

**Fig. 2:**
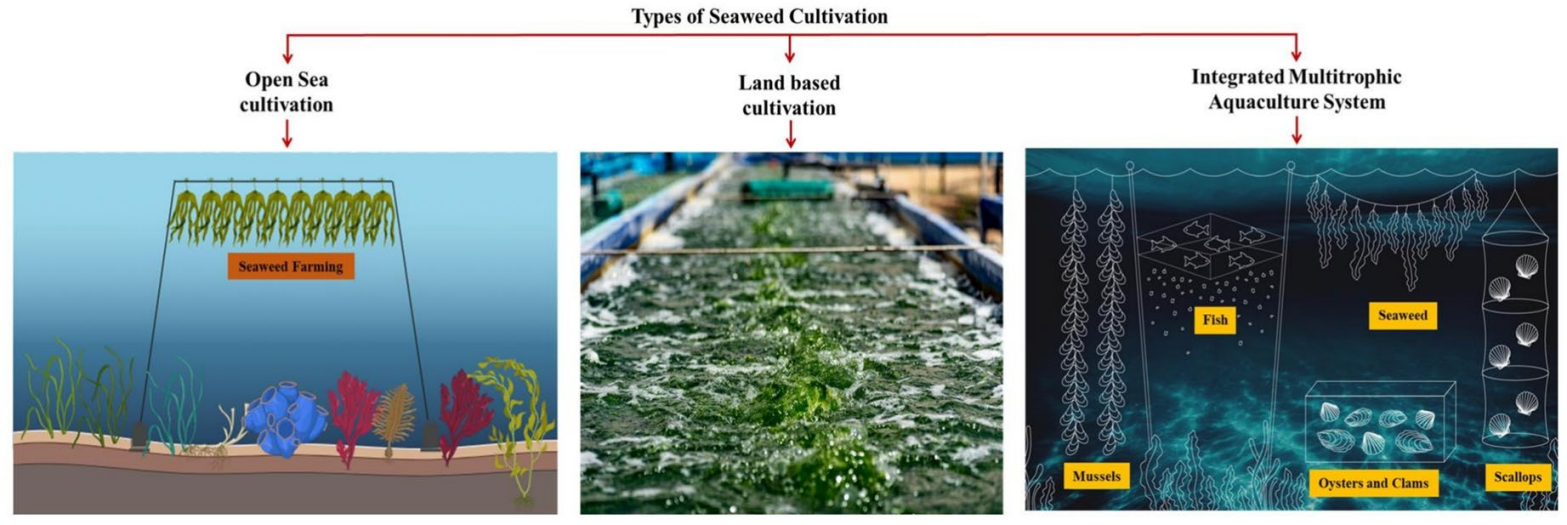
Various methods used for the cultivation of seaweed

It is essential to create an effective seaweed culture system to satisfy the growing demand for economically valuable seaweeds worldwide without over-exploitation and destroying marine ecosystems. Seaweed can be grown in open waters (offshore and nearshore), on land, and even in integrated multi-trophic aquaculture systems (Fig. 2).

### Open sea cultivation

This type of farming is seen in East Africa, Asia, and some parts of the US that including farming of seaweed at a distance from the coastline, where optimal state such as nutrients, temperature, water, and light, and movement are available. The seafloor can support seaweed growth adhering to a hard substrate or suspended on ropes, nets, or lines that float (Kim et al. 2017; Amosu et al. 2013; Buschmann et al. 2017). *Pyropia/Porphyra spp*., *Kappaphycus alvarezii*, *Saccharina japonica*, *Undaria pinnatifida*, *Gracilaria/Gracilariopsis* sp. and *Eucheuma striatum* are the most cultivated species in the open sea (Kim et al. 2017). Despite being labor-intensive, open-sea growing systems are a popular cultivation method because they are reasonably inexpensive for seaweed installation, maintenance, and seeding (Fernand et al. 2017). Open recultivation has several disadvantages. The most important of them is the ability of seaweeds and structures to withstand extreme weather conditions, including wind, waves, and tides. These cultures are extensively diversified and established in diverse geographic regions to mitigate environmental hazards and maintain economic sustainability (Hafting et al. 2012).

### Land-based cultivation

To achieve ideal growth conditions, land-based cultivation systems like ponds, tanks, or raceways supply clean seawater and carefully control lighting, temperature, and levels of fertilizer (Fernand et al. 2017; Mendoza et al. 2018). Globally, land-based seaweed farming can be built. It should be adjacent to the processing and manufacturing facilities as well as the water provider to provide a consistent supply of seawater and favourable growth conditions (Levy et al. 2008). The important benefits of land-based farming or cultivation include the flexibility to produce many products by simply altering nutrient inuts and cultivation conditions. This cultivation use the same facilities for growing different seaweed species, and precisely control the productivity and quality of the cultures without relying on genetically modified species (Fernand et al. 2017; Levy et al. 2008). Several genera, such as *Ulva*, *Chondrus*, *Palmaria*, and *Porphyra*, are frequently grown in land-based systems. The variety of goods offered in the worldwide seaweed market is anticipated to increase, nevertheless, as these technologies are used and modified for other seaweed species (Craigie et al. 2019; Demetropoulos et al. 2004; Grote et al. 2019; Fernand et al. 2017).

### Integrated multitrophic aquaculture system (IMTA)

A useful technique that can be applied to both land-based and open-sea environments is the Integrated Multitrophic Aquaculture system, which combines extractive aquaculture (such as shellfish and algae) with cage-fed animals (such as fish or shrimp) (Kim et al. 2017). The efficiency of these systems depends on seaweeds' ability to absorb and transform released CO_2_, phosphate, and ammonium into biomass (García-Poza et al. 2020). This technology cleans and filters the effluents, which can then be recycled back into the ponds or easily disposed of without causing environmental harm, promoting a more balanced ecology (Abreu et al. 2011). By converting the waste from primary cultured species into byproducts like fertilisers, food, and energy. IMTA can boost long-term sustainability and profitability while simultaneously increasing the commercial and environmental value of the cultivation (Turan et al. 2010).

## Methods used worldwide for seaweed farming

Traditional methods of harvesting seaweeds from wild or natural populations led to an overuse of marine resources. This problem is being addressed by the introduction of novel farming methods that are domesticating native species (Dalton et al. 1994). Many elements affect the effectiveness of large-scale seaweed cultivation, including the regeneration of thallus and its relation to environmental parameters like light, temperature, nutrients, and water flow (Tiwari and Troy 2015). It is the taxonomical traits of the seaweed species that determine the growing technique. While some species (like *Kappaphycus*, *Eucheuma*, *Gracilaria*, and *Chondrus*) require a single-step farming method through vegetative propagation. Other seaweeds thatpropagated from spores (like *Undaria*, *Laminaria*, *Enteromorpha*, and *Porphyra*) can’t survive through vegetative propagation and need a two-step or multi-step approach for its cultivation. Based on seaweeds’sorganizational structure (e.g., clonal vs. unitary organism), these are usually cultivated using one or different crops (Santelices 1999). Clonal species like *Kappaphycus*, and *Gracilaria* which are fragmented and vegetatively reproduced, rapidly grow directly in culture systems. Seaweed farming uses a variety of techniques, from large open-sea farming systems with long lines or rafts to intensive systems like tanks or ponds (Sahoo and Yarish 2005). In a study it was revealed that *conchocelis* culture holds the potential to revolutionize *Pyropia* sp. farming and the generation of seedling (Zhong et al. 2016). *Conchosporangia* production, vegetative growth, and conchospore releases are the three main phases of *Conchocelis* development.These two seaweeds are influenced by environmental factors (Wang et al. 2008). For industrial seedling production, it is essential for maintaining optimal culture conditions for control the growth and reproduction of species-specific *Conchocelis* cultures. Furthermore, the gametophyte (male) of *Palmaria palmata* has been successfully mass-propagated in tank cultures using sporelings generated from scattered tetra spores (Schmedes et al. 2020). With a special emphasis on boosting the economic potential of seaweed resources and to demonstrate the totipotency of red algae, seaweed tissue culture uses medullary tissue explants from the gametophytic (female) branches of *Chondrus crispus* Stackhouse (Garbary and Galway 2019). Callus culture, direct regeneration, and protoplast culture are different techniques used in seaweed tissue culture (Jiksing et al. 2022).

Direct regeneration produces less soma clonal variation than callus culture, so it is favoured for producing plantlets that are true to type (Yong et al. 2014b). For the clonal multiplication of seaweeds and production of large number of genetically identifiable seedlings for growth, this kind of tissue culture is perfect. The ability of *Gracilariales*, *Gelidiales*, *Halymeniales*, *Ceramiales* and *Gigartinales* to produce adventitious branches from explants have been demonstrated in numerous studies (Jiksing et al. 2022). Such tissue culture can be used to clonally propagate seaweed. It is usefulto provide propagated seaweeds to produce multiple identical seedlings or cultures. Direct regrowth has been effectively used in Provassoli enriched seawater (PES) or f/2 liquid media to generate homogenous seedlings from various species, including *Saragassum polycystum* (Muhamad et al. 2018), *Grateloupia filicina* (Baweja & Sahoo 2009) and *K. alvarezii* (Yong et al. 2014b) These seaweeds were cultivated to produce numerous bioactive compounds for wide range of industrial application (Yong et al. 2014b).

The plants that grew from callus culture has the main advantage of potentially producing novel genetic variations. It includes many plants with faster development rates, as a result of soma clonal variation (Charrier et al. 2015). To report this benefit, Reddy et al. (2003) compared the growth rate of micro propagules with regrowth from *K. alvarezii* callus with those grown in India. According to the reported studies, the micro propagules that emerged from the callus grew more quickly than the samples that were grown traditionally. Several factors, such as temperature, light, plant growth regulators (PGRs) like auxin and cytokinin have been used during the tissue culture process, affect the formation of callus on seaweed explants (Uji et al. 2016). Plant cells that alive but lack a cell wall are known as protoplasts. They have been thoroughly examined in a variety of fields, including protoclonal variation, somatic hybridization, metabolomics and proteomics, as well as cybridization and protoclonal variation research (Reddy et al. 2008a). Due to their totipotentcy, plant cells can generate multiple protoplasts, which can then be developed into seaweed seedlings (Huddy et al. 2013). By creating consistent seedlings for cultivation in a lab, this technique maintains natural seaweed populations. Furthermore, protoplast culture provides the advantage of modifying seaweed genetics by somatic hybridisation (Charrier et al. 2015). Chemically, polyethylene glycol and physically cell fusion electrical device have been used to induce protoplast fusion. Recently, research to create new hybrid strains using this somatic hybridisation technique is expanding. This technique has advantageous traits and are hard to achieve through conventional sexual reproduction, disease resistance, increased colloid yield, quality, and stress tolerance (Jiksing et al. 2022). Seaweed cultivation is practiced globally, although in recent years, nations such as China, the Philippines, Japan, Indonesia, Korea, and Malaysia have dominated the industry (Behera et al. 2022). Seaweed cultivation is currently experiencing a technological revolution, transitioning from conventional vegetative propagation to sophisticated tissue culture and hybridization. However, lack of in-depth ecological understanding, infrastructure accessibility, and regional equity continue to limit its worldwide potential. Therefore, future research on sustainable intensification, genetic innovation by maintaining biosafety, and inclusion in technology distribution can be useful for enhanced seaweed farming.

## Industrial applications

**Fig. 3:**
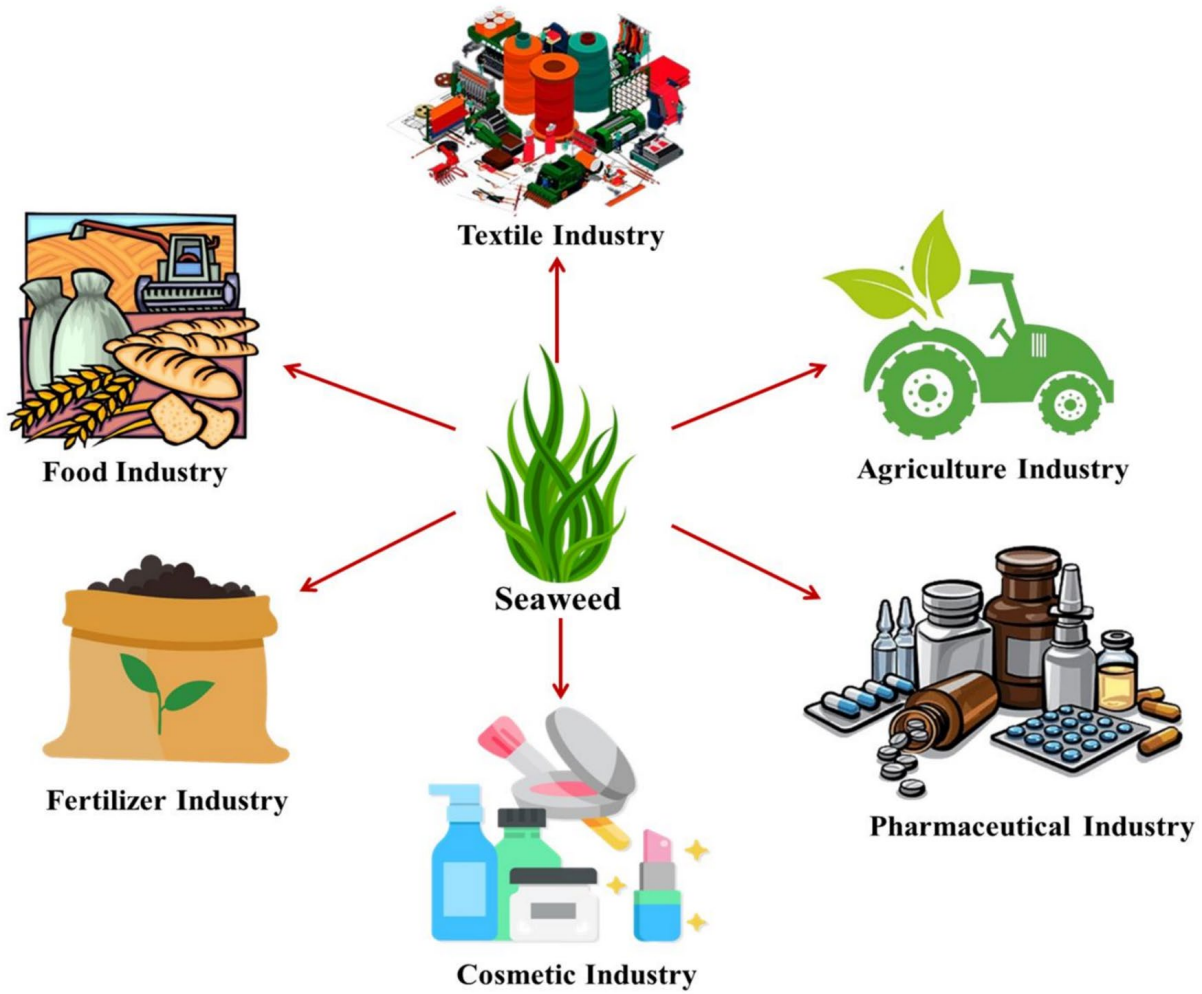
Various Industrial applications of seaweed

The pharmaceutical, nutraceutical, and functional food industries have all seen significant growth in recent years (Fig. 3). The general public is interested in the manufacturing of goods from seaweeds because they may offer health advantages beyond simple nourishment. As consumers become more aware of foods promoting health, research on dietary supplements is gaining popularity globally (Blikra et al. 2021). Seaweed is utilized to produce a vast array of beneficial industrial compounds. Proteins, lipids, polysaccharides, minerals, enzymes, trace elements, and essential vitamins are all abundant in it. It is also widely recognized for its therapeutic properties, nutritional value, and applications in pharmacology and other fields (Fig. 3). Additionally, it has multiple uses in the clinical and industrial sectors for the absorption of heavy metal contaminants. Furthermore, protein, vitamins, fiber, minerals, carbohydrates, and essential fatty acids are abundant in seaweeds. Seaweeds’ physiologically active chemical composition and quantity vary depending on their geographic location, ecological conditions, harvest season, and time of year (Abdel-Kareem and ElSaied 2022). Numerous bioactive compounds have been investigated as a novel source from seaweeds and their extracts, including carrageenan, polyphenols, polyunsaturated fatty acids and fucoidan (Choudhary et al. 2021). These secondary metabolites have many positive effects, including antitumor, antimicrobial, antidiabetic, anti-inflammatory, anti-aging, antiviral, and anti-obesity effects. Seaweeds have numerous applications in various biomedicine, cosmetics, pharmaceuticals, dermatology, and agriculture industries (Wijesinghe et al. 2012).

### Seaweed in the development of sustainable food industries

The demand for the seaweed market has expanded in recent years Advancements in seaweed based functional foods and increasing public awareness about dietary sources have contributed to a growing focus on health-conscious food eating practices (Gullón et al. 2021). The market for edible seaweeds is well-established in Asia**.** Seaweed is a prime food in many Asian nations and can be eaten fresh, and dried, asan alternative to vegetables (Zhang et al. 2022). Now, there is a growing demand in Europe (Mendes et al. 2022). According to the estimation of the use of seaweed as a meat substitute in the United Nations (UN), the use could increase by 75% by the year 2050. Edible seaweed is commonly considered to be a healthy and environment friendly choice in the world as a dietary supplement, as seaweed reduces the use of animal protein and decreases the impact of meat production on the environment (Gullón et al. 2021). According to the United Nations Food and Agriculture Organization (FAO), seaweed is the ideal food for the year 2021 due to high content of protein and low levels of fat, sugar, and cholesterol. The primary obstacles in this expanding market of seaweeds are the absence of strong guidelines for cultivating or harvesting seaweed using natural resources for large-scale production, as well as the general awareness about the potential benefits of seaweed as an alternative to foods (Losada-Lopez et al. 2021; Moss et al. 2021). The intake of a person for the consumption of seaweed as a supplement is affected by several factors, like its flavour, texture, aroma, its advantages and disadvantages, its cost, and accessibility. Redway et al. (2022), have reported that 46% of people in the UK are unaware of the health advantages of seaweed. Some nations are making quicker progress on the use of seaweeds as food. For example, several research and innovation programs and projects have been promoted in Chile to increase the use of seaweeds (Basically *Ulva* sp., green seaweed, *Durvillaea incurvate,* and *Chondracanthus chanmissoi*) to develop new food products, supplements, and recipes (Rogel-Castillo et al. 2023). The uses of Chilean seaweed for human consumption were encouraged people to include seaweed in their diets. Similarly, the use of seaweed from the Los Lagos region of Chile have been used in the production of new food products. Seaweeds like *Porphyra sp*., *Durvillaea antarctica*, *Chondracanthus chamissoi*, and *Callophyllis variegata* are used for the production of foods such as energy bars, Snacks, and other food products (Rogel-Castillo et al. 2023). Nowadays reformulated sausages (frankfurters) are made using *Undaria pinnatifida*, *Himanthalia elongata, Palmaria palmata* and *Porphyra umbilicalis* in place of adding salt during their preparation. According to Vilar et al. (2020), the amount of added salt and fat content of the pork was lowered by 21% and 50% by adding the red and brown seaweeds (Vilar et al. 2020). Moreover, these seaweeds have also added more moisture, a darker colour, a different texture, protein, and less ash as compared to the ordinary frankfurters. These frankfurters were less chewy and softer as well. These frankfurters were nutritious by the addition of seaweeds such as *U. pinnatifida*, *P. umbilicalis*, *P. palmata*, and particularly *H. elongata*. According to Yuan et al. (2023), *H. elongata* was useful in the preparation of the most promising frankfurters, but further recipe modification is required to add other benefits to these foods. Seaweeds are the new source of natural pigments for use as food colouring. In Northern Spain, 20 brown and 4 red seaweeds have been reported to contain chlorophyll and fucoxanthin (a carotenoid) content. The seaweeds *U. pinnatifida* contained the highest concentrations of fucoxanthin pigments which makes them appropriate for use as natural food colouring (Rubiño et al. 2022). Seaweeds possess the potential to revolutionize the sustainable food industry by providing nutritional, environmental, and functional advantages. However, their complete integration into global food systems is still hampered by information gaps in consumer behaviour, regulation, sensory qualities, and product design. Future study on integration of marine biology, food science, social science, and policy frameworks can be useful for sustainable use of seaweed in food industries.

### Seaweeds in the manufacturing of cosmetics

Seaweed is a beneficial marine alga for cosmetic compositions due to its bioactive compounds, which have several characteristics (Jesumani et al. 2019; Couteau et al. 2020; Alhajj et al. 2020). A range of cosmetics, including creams, lotions, and ointments, contain bioactive ingredients (including vitamins, minerals, and antioxidants) that support the health of the skin, hair, and nails (Couteau et al. 2020, 2016; Kim et al. 2014). These cosmetics are useful to enhance appearance and purify the skin without causing damage to the user. These seaweeds can be formulated for the production of various lotions or creams and edible substitutes like pills or functional foods with cosmetic benefits (Kim et al. 2014). Moreover, these cosmetics have anti-lipidemic and antioxidant, antimicrobial, anticancer, and anti-allergic properties (Cheong et al. 2018; Pallela et al. 2010; Fernando et al. 2017; Liu et al. 2018). Carbohydrates, vitamins, minerals, amino acids, phenolic compounds, proteins, peptides, sterols, and lipids (polyunsaturated fatty acids) are all found in seaweed. These compounds present in seaweeds are used as active components due to their strong beneficial effects (Jesumani et al. 2019; Pallela et al. 2010; Freitas et al. 2020). Now, various cosmetics companies use compounds and extracts of seaweed in their products. These can be used as active substances, moisturizers, thickeners, gelling agents, colours, preservatives, additives, or aroma components (Lourenco-Lopes et al. 2020). Cosmetics can also be made by combining seaweed extracts from different species. As an example, Aroma shoppe seaweed Gel (Ian Benham Cosmetic, Winschoten, The Netherlands) and Sealgae (Lusalgae, Figueira do Foz, Portugal). *Ecklonia kurome* can be extracted using a particular method to extract the bioactive compound phenol tannins; the result is ECKLEXT^®^ BG (NOF group, Tokyo, Japan). Chlorofiltrat^®^ Ulva HG is mostly composed of hydroglycolic extracts from the green algae *Ulva lactuca* (CODIF, Saint-Malo, France). Also, seaweed-derived substances such as mycosporine-like amino acids from *Porphyra umbilicalis* (red seaweed) are found in sunscreen products like Helionori^®^ (Gelyma, Marseille, France), Helioguard^®^ 365 (Mibelle biochemistry, Buchs, Switzerland) and Aethic Sôvée^®^ (Which uses photamin, an extract rich in Phenolic compounds, marketed by AETHIC^®^ in London, UK) (Siezen 2011; Cardozo et al. 2007; Morais et al. 2021). The extracts of *Chondrus crispus*, which is rich in sulfated polysaccharides, are made into Gelcarin^®^ (Dupont Nutrition and Bioscience, Wilmington, DEL, USA). The specific extracts function as a gelling, thickening, and stabilizing ingredient in cosmetic products (Morais et al. 2021). They are widely used in the cosmetic industry, but since they are a natural resource that comes from the wild, it’s important to assess the ecological impact and ensure sustainability. This is necessary to maintain the balance of the ecosystem and ensure cost-effectiveness on a larger, industrial scale.

### Agricultural industries

Different kinds of seaweeds are nowadays used in agricultural sectors for the production of organic fertilizers (López-Mosquera et al. 2011). *Chondracanthus squarrulosus* has been used as an agricultural fertilizers formulation by culturing it under semi-controlled conditions to enhance biomass production (Pacheco-Ruiz et al. 2004). *Kappaphycus alvarezii* one of the red seaweeds, is used as thickening and stabilizing product formulation as it contains carrageenan. This seaweed contains large amount of potassium, growth hormones, other micro- and macronutrients, which improves crop yields from 15 to 40%. *K alvarezii* has been used as animal feed formulation. This seaweed has got popularity and importance as agricultural product and animal feed in India (Seth and Shanmugam 2016). In agricultural industries, *Saccharina japonica*, *Kappaphycus, Eucheuma, Porphyra*, *Undaria pinnatifida*, *Pyropia* species, and *Gracilaria* have been used for the production of fish feeds (Seth and Shanmugam 2016). *Fucus*, *Ascophyllum*, *Laminaria*¸ *Sargassum* has been used in the formulation of biofertilizer (Dhargalkar and Pereira 2005). *A. nodosum* has been used as biostimulant as it produces plant growth stimulating hormone gibberellic acid (GA_3_) (Akazawa et al. 1985). Human activity is the cause of water contamination in agricultural drains. High concentrations of naturally occurring chemicals or the introduction of artificial substances (xenobiotics) into the environment cause this problem. Organic compounds released by domestic, agricultural activities cause pollution in the form of inorganic materials (Mouchet 1986). These contaminants are treated by applying the algal extractsto determine their effectiveness. Direct application to the soil, foliar spraying, pellet coating of seeds as a post-harvest treatment, or a combination of these techniques are useful for effective production. One of the most popular approaches among these is the combination of foliar spraying and soil treatment (Grzesik et al. 2017; Khan et al. 2009; Hong et al. 1995; López et al. 1999; Povero et al. 2016). This combo technique supplies the soil with essential nutrients that encourage healthy seed germination, plant emergence, and early growth. Foliar treatment enhances plant growth, both vegetative and reproductive, resulting in bigger yields and better harvests (Lopez-Padron et al. 2020). Numerous studies recommend sustainable farming practices to alleviate the scarcity of arable land and enhance urban food security. Methods like hydroponics have become more popular because they can produce fresh products with superior quality by using fewer resources. Traditional hydroponic systems that rely on chemical nutrients may have negative consequences on the environment, including resource depletion and eutrophication. Despite numerous advantages of sea weeds, numerous barriers prevent seaweed products from used in agriculture, including its variability by season and location, high expenses for production and processing, uncertainties in regulations and the absence of uniform quality standards. Future research on barrier reduction can be useful to overcome the existing challenges.

## Health benefits of the seaweed

**Fig. 4:**
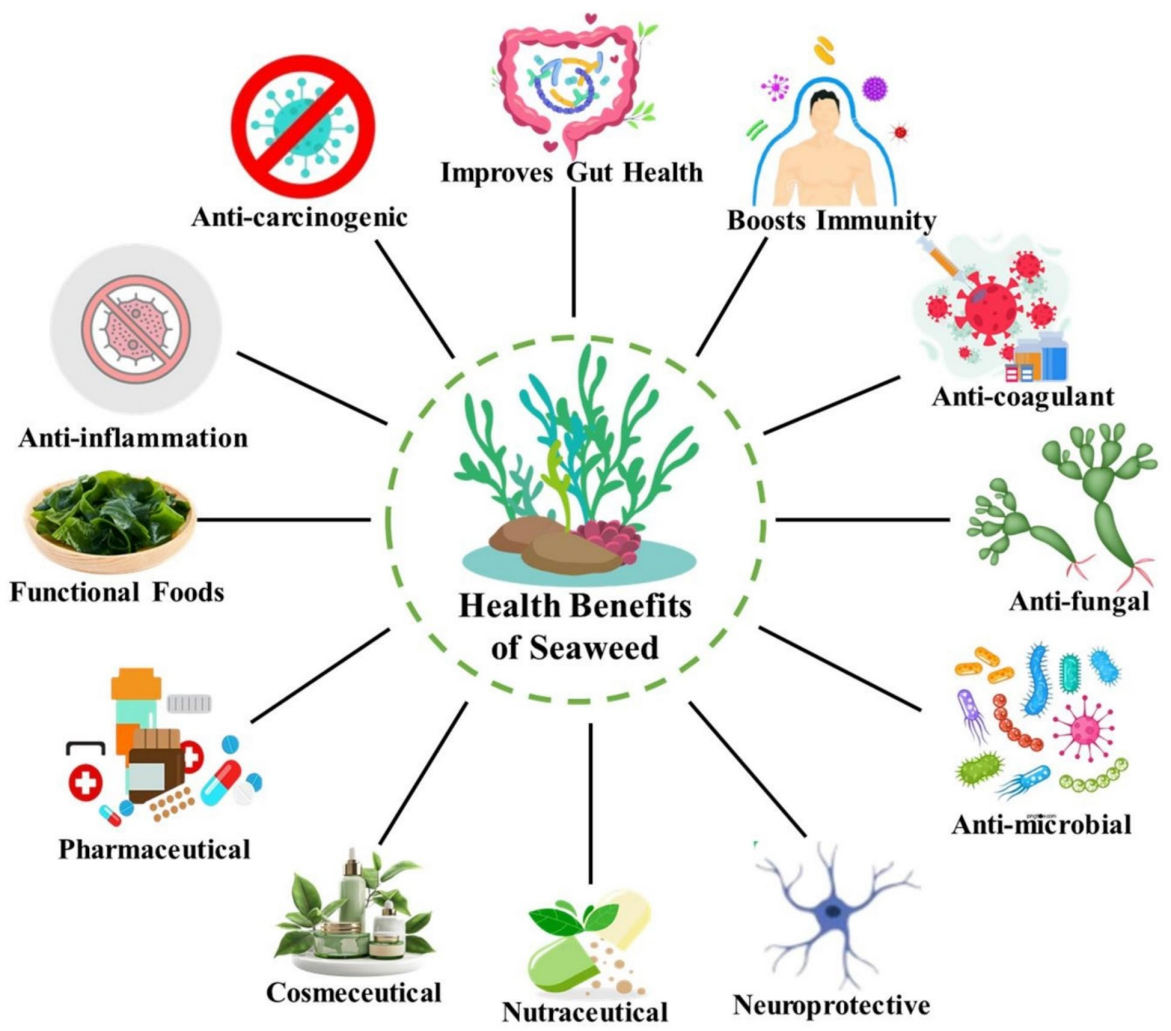
Important Heath benefits of seaweed as functional food

Seaweed has numerous health benefits as it contains minerals (Ca, Fe, I, Mg, P, K, Zn, Cu, Mn, Se, and F), vitamins (A, B1, B2, B9, B12, C, D, E, and K), dietary fibres, protein, essential amino acids, and polyphenols (Lomartire et al. 2021). Edible seaweed species like *Codium*, *Gracilaria*, *Ulva*, and *Acanthopora* include a greater levels of dietary fibre ranging from 23.5% to 64% DW (dry weight), where polysaccharides are greater than wheat bran (Mamat et al. 2016). The edible seaweeds ulvan (*Chlorophyta*), fucoidan (*Phaeophyta*), and carrageenan (*Rhodophyta*) include sulphated polysaccharides (SPS), which have a broader range of uses in the pharmaceutical, nutraceutical, and cosmeceutical industries. These SPS also possess antioxidant, anticancer, neuroprotective, anti-inflammatory, mental-health, bone-health, antidiabetic, UV protective, anticoagulant, dyslipidaemia, immunomodulatory, anti-HIV activities, and heart-health (Fig. 4) (Stephen et al. 2017; Nagarajan and Mathaiyan 2015; Kumar et al. 2018). A recent study revealed that SPS is a more effective than synthetic antioxidants such as butylated hydroxytoluene and butylated hydroxyanisolenitric oxide at scavenging nitric oxide (Mamat et al. 2014; Sivaraman et al. 2023). Rajapakse and Kim (2011) investigated the impact of consumption of seaweed on human digestive health and revealed that the dietary fibres present in seaweeds reduced the risk of colorectal cancer and suppressed inflammation in the gastrointestinal tract. Global epidemiological research shows that the health risk and illness are much lower in nations where seaweed is regularly consumed (Kumar et al. 2018). Apart from polysaccharides, seaweeds also contain higher protein content than terrestrial plants like soybean, pulses, etc. (Dhargalkar 2015). Glutamic acid, taurine, threonine, arginine, alanine, and aspartic acid are frequently recorded amino acids found in some seaweeds; however, some seaweeds like *C. crispus*, *Gracilaria* sp., *O. pinnatifida*, and *Porphyra* sp. have not contained phenylalanine and methionine amino acids (Urbano and Goni 2002). The seaweeds such as *A. nodosum, F. vesiculosus*, and *Ulva* sp. contain 6.47–24% of the essential amino acids in the concentration of 34.4 g/100 g, 25.1 g/100 g, and 27.0 g/100 g protein, respectively. As a result, this quantity is comparable to the RDI (recommended dietary intake) for dietary protein for adults (Abirami and Kowsalya 2012). As edible seaweeds contain a wide range of essential amino acids, which arecomparable with plants such as legumes and soybeans, theyare regarded as an alternative to animal proteins (Murata and Nakazoe 2001). *Ulva*, *Acanthophora*, and *Gracilaria* species have high levels of omega-3 fatty acids (Abirami and Kowsalya 2012), which have been extensively studied for their anti-inflammatory, antihypertensive, and antihyperlipidemic properties, as well as their ability to block the angiotensin I-converting enzyme. It has been demonstrated that lutein and zeaxanthin derieved from Seaweed offer protection against macular degeneration. These dietary pigments have unique metabolic activities in human health and are very beneficial nutraceutical component (Murata and Nakazoe 2001). They are strongly linked to anticancer, anti-hypercholesterolemic, and neurodegenerative diseases and have potent antioxidant activities (Teas et al. 2013). Seaweeds have a diverse spectrum of secondary metabolites and are always of interest to scientists due to their potential bioactivity when compared to land plants. Alkaloids, flavonoids, carotenoids, polyphenols, and phlorotannins have all been demonstrated to have hypoglycemic properties. Evidence suggests that fucoidan can serve as an anti-proliferative agent by increasing dendritic cell maturation, in conjunction with other cytokines, and modulating the immune system of humans (Lowenthal and Fitton 2015). Bioactive substances from seaweed have been discovered to be safe and beneficial against type 2 diabetes by reversing carbohydrate metabolism enzymes (Abirami and Kowsalya 2013). Phlorotannins, gallic acid, quercetin, phloroglucinol, carotenoids, and their derivatives are the frequently investigated phytochemicals in seaweeds. All genera of seaweeds have been found to include polyphenol compounds; however, their prevalence is most likely highest in brown and red seaweeds (Abirami and Kowsalya 2013). Seaweed including *Saccharina cichorioides*, *Fucus evanescens*, and *Undaria pinnatifida, which generate* fucoidan, significantly reduces the proliferation of human colon cancer cells (DLD-1) and showed decreased cytotoxicity against normal mouse epidermal cells (JB6 C141) (Ermakova et al. 2011). The isolated fucoidan from *E. cava*, *S. hornery*, and *C. costata* seaweed demonstrated comparable cell proliferation against colon cancer cells (SK-MEL-28), melanoma (DLD-1) in humans (Ermakova et al. 2011; Ale et al. 2011). Additionally, epidemiological research showed that eating seaweed reduces the risk of endometrial, ovarian, and breast cancer in Japanese people when differentiated to people in other countries (Murata and Nakazoe 2001). After therapy for 4 weeks (2% Undaria lipid-fed rats), the brown seaweed *U. pinnatifida* decreased white adipose tissue (WAT) in Wistar rats and KK-Ay mice. It was intriguing to learn that there was a significant decrease in body weight without a change in the food intake of rats (Yoshinaga et al. 2018). Likewise, when administered to C57BL/6 J mice, *U. pinnatifida* extract lowers plasma leptin levels and epididymal adipose tissue (Ale et al. 2011). When compared to obese rats, this extract's offucoxanthin significantly decreased adipocyte size, fasting blood glucose, and levels of insulin (Gammone and D'Orazio 2015). Other species, such as *L. japonica* and *L. ochotensis* that contain fucoxanthin, demonstrated anti-obesity effects on mice and to inhibit absorption of fat and serum triglyceride levels in vivo (Kang et al. 2012). Regular use of seaweed, whether in functional foods, supplements, or as part of a balanced diet, can have a substantial impact on overall health. However, moderation is essential, as excessive iodine intake might harm thyroid health. With its multiple benefits, seaweed is an excellent supplement to a sustainable and health-conscious diet.

## Challenges and future prospects

The research on seaweed and its industries are facing numerous environmental hazards like climate change, the high cost in sustainable cultivation and processing. Furthermore, the substantial expense associated with seaweed cultivation and its processing technologies continues to be a significant impediment, particularly in regions that lack technical expertise or infrastructure. Inconsistent international rules and overexploitation of wild resources also impede the expansion of seaweed cultivation. The absence of clear and consistent guidelines frequently results in the unregulated exploitation of natural seaweed resources and causes a threat to the long-term ecological balance and marine biodiversity. Furthermore, poor public awareness, weak market infrastructure, and full commercialization of seaweed-based products. However, innovative developments in biotechnology, remote sensing, and molecular biology have ability to improve bioactive component extraction, culture efficiency, and species identification. Seaweed farming can be made even more valuable ecologically and economically by combine climate mitigation techniques like carbon sequestration. Cooperative international initiatives and implementation of advance techniques can be useful in reduction of waste and utilization of seaweed resources to reach their full potential.

## Conclusion

Seaweed is an important marine resource with a variety of ecological, nutritional, and industrial uses. Its botany displays distinct adaptations and morphological features that enable survival in a variety of maritime habitats. Understanding seaweed distribution emphasises the need for conservation in the face of concerns like as change of climate and habitat demolition. Despite extraction and standardisation issues, its diverse chemistry, which includes bioactive chemicals, has vast potential for medicines, nutraceuticals, and industrial applications. Advances in cultivation and harvesting techniques, such as integrated multitrophic aquaculture and offshore farming, are opening the road for long-term large-scale outcomes.

## Data Availability

All data generated or analyzed during this study are included in this article.
